# Unveiling the transcriptomic landscape and the potential antagonist feedback mechanisms of TGF-β superfamily signaling module in bone and osteoporosis

**DOI:** 10.1186/s12964-022-01002-2

**Published:** 2022-11-28

**Authors:** Ying-Wen Wang, Wen-Yu Lin, Fang-Ju Wu, Ching-Wei Luo

**Affiliations:** Department of Life Sciences and Institute of Genome Sciences, National Yang Ming Chiao Tung University, 155 Li-Nong Street, Section 2, Beitou, Taipei, 112 Taiwan

**Keywords:** Antagonist, BMP8, Mesenchymal stem cell, Osteoporosis, TGF-β superfamily

## Abstract

**Background:**

TGF-β superfamily signaling is indispensable for bone homeostasis. However, the global expression profiles of all the genes that make up this signaling module in bone and bone-related diseases have not yet been well characterized.

**Methods:**

Transcriptomic datasets from human bone marrows, bone marrow-derived mesenchymal stem cells (MSCs) and MSCs of primary osteoporotic patients were used for expression profile analyses. Protein treatments, gene quantification, reporter assay and signaling dissection in MSC lines were used to clarify the interactive regulations and feedback mechanisms between TGF-β superfamily ligands and antagonists. Ingenuity Pathway Analysis was used for network construction.

**Results:**

We identified *TGFB1* in the ligand group that carries out SMAD2/3 signaling and *BMP8A*, *BMP8B* and *BMP2* in the ligand group that conducts SMAD1/5/8 signaling have relatively high expression levels in normal bone marrows and MSCs. Among 16 antagonist genes, the dominantly expressed TGF-β superfamily ligands induced only *NOG*, *GREM1* and *GREM2* via different SMAD pathways in MSCs. These induced antagonist proteins further showed distinct antagonisms to the treated ligands and thus would make up complicated negative feedback networks in bone. We further identified TGF-β superfamily signaling is enriched in MSCs of primary osteoporosis. Enhanced expression of the genes mediating TGF-β-mediated SMAD3 signaling and the genes encoding TGF-β superfamily antagonists served as significant features to osteoporosis.

**Conclusion:**

Our data for the first time unveiled the transcription landscape of all the genes that make up TGF-β superfamily signaling module in bone. The feedback mechanisms and regulatory network prediction of antagonists provided novel hints to treat osteoporosis.

**Video Abstract**

**Supplementary Information:**

The online version contains supplementary material available at 10.1186/s12964-022-01002-2.

## Background

Bone is a metabolically active organ that remodels itself constantly throughout life [1, 2]. To maintain bone homeostasis, the fluctuation between bone formation and bone resorption must be precisely controlled and the cells in the osteogenic lineage are now known to be responsible for the balance between these two processes [3]. Generally, mesenchymal stem cells (MSCs) and their osteogenic lineage cells secrete multiple signaling factors that direct the commitment of MSCs and their subsequent osteogenic development. These factors are later immobilized together with the bone matrix during mineralization. Interestingly, it is believed that the MSC-derived cells, such as osteoblasts and osteocytes, further couple the initiation of oteoclastogenesis by secreting cytokines such as receptor activator of nuclear factor kappa-B ligand (RANKL), which then stimulate hematopoietic stem cells to give rise to osteoclasts [4, 5]. Along with the osteoclast-mediated bone resorption, the matrix-embedded osteogenic factors are again released and this creates a microenvironment favoring for the next run of bone formation [6, 7].

Based on the above, it is widely accepted that MSCs and their differentiated linages play dominant roles in controlling the initiation and dynamic balance during bone remodeling via the local release of various signaling molecules, in which the TGF-β superfamily ligands are emerging as the key modulators. The TGF-β superfamily ligands consist of more than 35 members in vertebrates and are the most abundant cytokines in bone [8–10]. They exert their effects via binding to receptor complexes formed by various type I and type II serine/threonine kinase receptors. Depending on the receptor specificity to different receptor-associated effector SMADs, these ligands can selectively mediate either SMAD2/3 signaling or SMAD1/5/8 signaling [8, 11]. Intriguingly, although coupling with the common mediator SMAD4 to convey their signals to the nucleus, the activated SMAD2/3 and SMAD1/5/8 complexes exert different functions and these can even counteract each other mutually in bone homeostasis. Generally, activation of SMAD2/3 signaling, the canonical downstream of TGF-βs and activins, promotes chemotaxis, proliferation, and early osteogenic differentiation of MSCs. However, it in turn can inhibit osteoblast maturation, mineralization, and transition into osteocytes. By contrast, SMAD1/5/8 signaling is normally activated by BMPs, and this then initiates, promotes, and maintains chondrogenesis and osteogenesis [12, 13]. This means that the identities as well as the temporospatial amounts of the TGF-β superfamily ligands expressed in the bone microenvironment will determine the seesaw balance between SMAD2/3 pathway and SMAD1/5/8 pathway, which in turn reflects osteogenic efficiency.

The intensity of above SMAD signaling in bone is not only controlled by the TGF-β superfamily ligands, but it can also be further modulated by the local profile of other molecules involved in the signaling module; these include various receptors, SMADs and also a group of secreted antagonists. These antagonists can bind directly with their selective TGF-β superfamily ligands and thus preclude the interaction of these ligands with their surface receptors [14]. Currently, over 16 agonist members have been identified and they can be classified into the NBL1 subfamily, TWSG1, NOG (also named noggin), the chordin subfamily and the follistatin subfamily [15, 16]. Importantly, the expression of some antagonists can further be modulated in a synergistic manner by their targets or by other TGF-β superfamily ligands via the induced SMAD signaling [16–18]; this points to the need of local feedback mechanisms for the delicate balance between the TGF-β superfamily ligands and their antagonists. Taken together, one can imagine that the proteins involved in the modulation of TGF-β superfamily signaling form a highly complex and interactive network and that this then allows a fine-tuning of the intensities of different pathways as well as their corresponding responses in the bone microenvironment.

Currently, although several genes in the module of TGF-β superfamily signaling have been shown to feature prominently in multiple aspects of bone functionality, knowledge regarding the global views of this signaling module, such as the whole gene expression profiles and their interactive regulations, in the bone microenvironment or the progression of bone-related diseases are still lacking. Taking advantage of the rapid accumulation of a range of bone-related transcriptomic datasets, we here tried to unveil the expression status of all the genes involved in conducting TGF-β superfamily signaling in bone marrows and bone marrow-derived MSCs. The interactivity between the dominantly expressed TGF-β superfamily ligands and induced antagonists were also clarified in order to better understand the complicated negative feedback networks in bone. Using the above information, we further explored the involvement of this signaling module in the progression of primary osteoporosis.

## Material and methods

### Animals and ethics

C57BL/6 mice were purchased from the animal center of National Yang Ming Chiao Tung University. All animal experiments conformed to the Guide for the Care and Use of Laboratory Animals and were approved by the Institutional Animal Care and Use Committee of National Yang Ming Chiao Tung University (Permit Number: 1070108).

### Reagents and cytokines

Cell culture medium, fetal bovine serum and penicillin–streptomycin–glutamine were purchased from Invitrogen. SB431542, dorsomorphin and other chemicals unless noted were purchased from Sigma-Aldrich. Human BMP2 (355-BM), human TGF-β1 (240-B), human NOG (6057-NG-025), human GREM1 (5190-GR-050) and human GREM2 (8436-PR-050) proteins were purchased from R&D systems. Recombinant human BMP8A protein was generated as described previously [19].

### Isolation of mouse bone marrows and culture of bone marrow-derived MSCs

Bone marrows were isolated from mice (6–8 weeks old) as previously described [20]. In brief, after sacrifice, the femurs and tibias were dissected by carefully removing the connective tissues and attached muscles. The proximal and distal ends of the femurs and tibias were removed and the bone marrows were collected using centrifugation at 10,000*g* for 15 s.

To isolate the primary bone marrow-derived MSCs, the freshly isolated bone marrows were resuspended and plated on a petri dish using MesenCult Expansion medium (StemCell, 05513) to exclude the contamination of hematopoietic lineage cells. The following culture, passage and the expansion of the marrow-derived MSCs were according to the guideline of MesenCult Expansion Kit. Human bone marrow-derived MSCs were purchased from ScienCell (No. 7500) and cultured in the mesenchymal stem cell medium (ScienCell, 7501).

### Immunoblotting

The conditioned media from cultured mouse bone-marrow cells or MSCs were collected for detecting the existence of endogenous BMP8 protein. Briefly, the freshly isolated bone marrow cells described above were resuspended by serum-free DMEM and cultured at 5% CO_2_, 37 °C for 24 h. The conditioned medium was concentrated and quantified. A total of 20 μg protein was loaded per lane for subsequent immunoblotting analysis. C3H10T1/2 cells, a mouse MSC line, were first transfected with shRNA plasmid against GFP (control) or *Bmp8a* (target sequence CCTGCGTAAACACCGTAACAT) by Lipofectamine 2000 reagent followed by selection with puromycin (5 µg/ml). The selected cells were cultured in serum-free DMEM at 5% CO_2_, 37 °C for 24 h. The conditioned medium was concentrated for immunoblotting analysis and the cells were subjected to RNA extraction to detect the *Bmp8a* knockdown efficiency.

For Western blotting, the primary antibodies used in this study included antibodies recognizing BMP8 (R&D systems, AF1073), phospho-SMAD1/5/8 (Cell Signaling, 13820), total SMAD1/5/8 (Santa Cruz, sc-6031-R), phospho-SMAD2/3 (Cell Signaling, 8828), total SMAD2/3 (Cell Signaling, 8685), total SMAD1 (Santa Cruz, sc-7965), NOG (R&D systems, AF719), GREM1 (R&D systems, AF956), GREM2 (R&D systems, AF2069) and β-actin (Chemicon, MAB1501).

### cDNA preparation and real-time PCR quantification

For preparation of cDNA from treated MSCs, total RNA from each sample was extracted using the TRIzol reagent (Thermo Fisher Scientific) and then reverse-transcribed using the High-capacity cDNA reverse transcription kit (Applied Biosystems) with the oligo-dT primer. For subsequent real-time PCR quantification, the Power SYBR Green Master Mix reagent (Applied Biosystems) was used. The accession number and primer set of each gene assessed in this study were listed in Additional file 1. For all genes, single products were confirmed by melting curves and agarose gel electrophoresis; the no template control was applied and the Ct value remained un-determined. For relative quantification, the level of *Actb* in each prepared cDNA was used for normalization and the indicated gene levels relative *Actb* were calculated using the 2^(−ΔCt)^ formula. Amplification efficiencies for each gene amplicon were evaluated to be similar using serial dilutions of the cDNA samples [21]. To compare the expressional changes, the expressional level of each gene before treatment with TGF-β superfamily ligands was served as the onefold control for normalization.

### Luciferase reporter assay

Luciferase assays were performed as described previously [22]. Briefly, HEK293T cells were transfected with the reporter vector, either BMP responsive element (BRE)-luciferase or CAGA-luciferase, and pCMV-β-galactosidase vector in the ratio 10:1 in the 48-well plate for 6–8 h. After overnight treatment with ligands, the cells were lysed with 10X lysis buffer (10% Triton X 100, 10% glycerol, 250 mM Tris/pH7.4, 100 mM MgCl_2_, 20 mM EGTA, and 20 mM dithiothreitol) for luciferase assays. The luciferase intensity was normalized with the β-galactosidase activity.

### Characterization of transcriptomic datasets, network connection and statistical analyses

Relevant bone-related RNA-seq transcriptomic datasets were retrieved from the Gene Expression Omnibus (GEO) repository. The datasets derived from healthy human bone marrows include GSE94339 (n = 3; three females; 33–51 years old), GSE102312 (n = 7; two females and five males) and GSE120444 (n = 8; 24–60 years old). GSE94736 (n = 28; 16 young females at 28.7 years of mean age and 12 old females at 73.3 years of mean age) was selected as a dataset representing the transcriptome of normal bone marrow-derived primary MSCs. All the measured mRNA transcripts, including receptors, SMADs, ligands and antagonists, within the datasets were transformed from reads per kilobase of transcript per million mapped reads (RPKM) or fragments per kilobase of transcript per million mapped fragments (FPKM) into transcripts per million (TPM) for data integration and comparison [23]. For presenting the data with skewed distribution, the log_2_ transformation was chosen. As the normalized counts can be equal to zero or less than 1, which respectively leads to undefined or a negative value during log transformation, each value was further shifted by adding 1 pseudo-count into TPM + 1 before performing log_2_ transformation [24].

GSE35958 (human MSCs from osteoporosis: n = 4, 79–94 years old; human MSC from age-matched controls: n = 4, 79–89 years old) was selected as a dataset for analyzing the differential expression of genes in primary osteoporosis. To integrate a wide range of the expression value of different genes into a heat map, the expression value of each gene was converted into percentage by defining the lowest value and the highest value in each gene group as 0% and 100%, respectively. The expression pattern and hierarchical clustering were illustrated using MultiExperiment Viewer (MeV; https://doi.org/10.1093/bioinformatics/btr490). The GSEA software from Broad Institute (http://www.gsea-msigdb.org/gsea/index.jsp) was used to determine whether a defined set of genes shows significant differences between osteoporotic and healthy individuals. The gene sets of canonical pathways derived from the KEGG pathway database (C2-CP-KEGG) in Molecular Signatures Database (MSigDB) collections were chosen for analysis based on the GSEA method.

Ingenuity Pathway Analysis (IPA, April 2022 release; https://analysis.ingenuity.com/) was used to assess biological relationships among genes. The up-regulated antagonist genes were subjected to the causal network analysis in IPA to find possible regulators. Molecules were selected by setting the *P*-value of overlap below 0.05 as the threshold. Z-score above 2 was defined as the threshold of significant activation, whilst Z-score below − 2 was defined as the threshold of significant inhibition. The obtained regulators were then subjected to the analysis of overlay canonical pathways in IPA to visualize the potential pathways for the observed expressional changes.

For other statistical data analyses, student *t*-test was used to compare differences between two groups, whereas one-way ANOVA followed by Bonferroni post test (PRISM software version 5.01; GraphPad) was used to compare differences among multiple groups. For representative images of Western blotting, at least three independent experiments were performed and showed similar results.

## Results

### The transcriptomic landscape of the genes involved in TGF-β superfamily signaling module

**Fig. 1:**
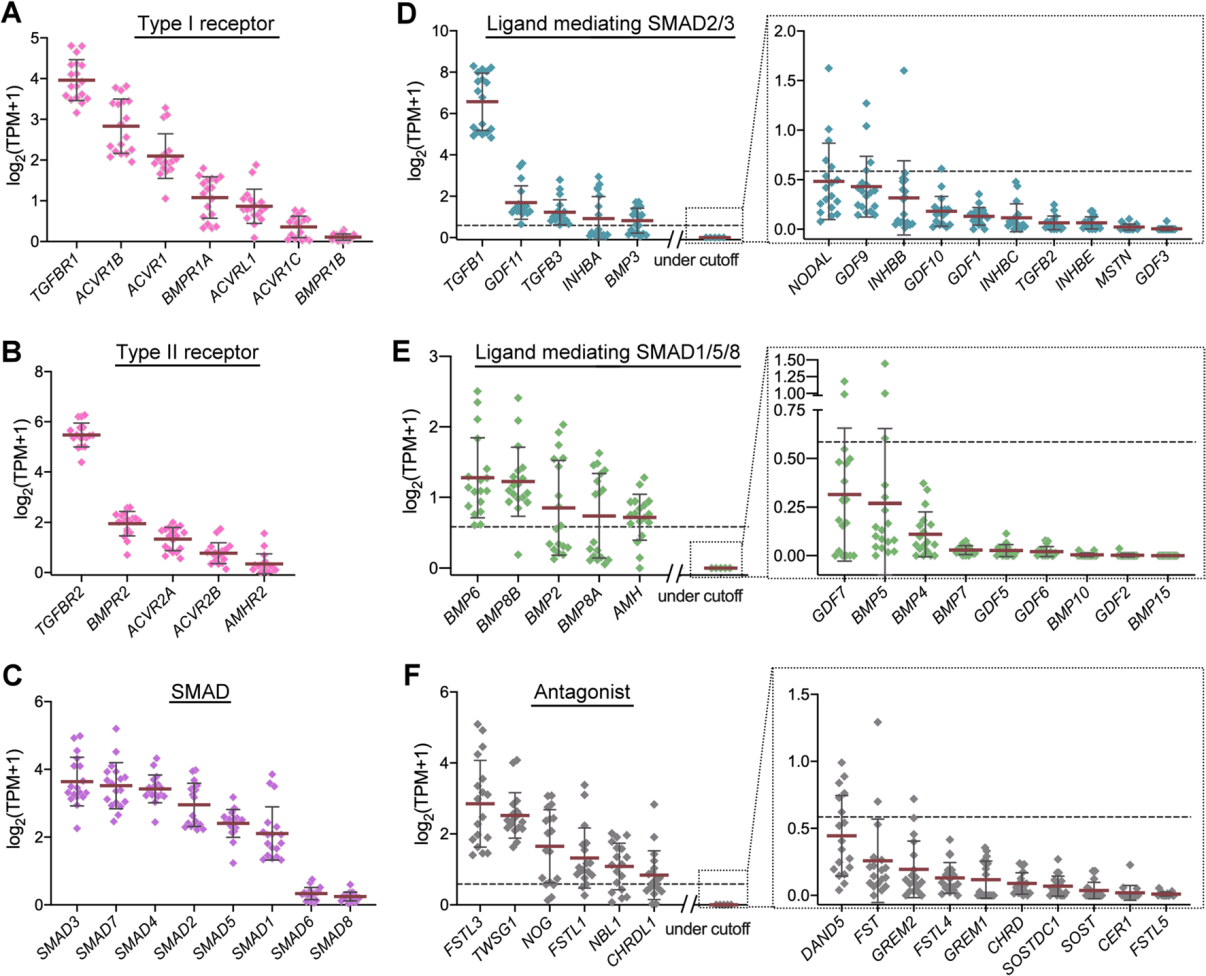
Transcriptomic landscape of the genes in TGF-β superfamily signaling module in human bone marrows. The transcripts of selected genes were extracted from transcriptomic datasets derived from healthy human bone marrows and converted into TPM for integration. The detailed TPM values for each gene were presented in Additional files 2 to 4. For the graphic presentation, the data were transformed to log-space by taking log_2_ (TPM + 1) (as described in Materials & Methods). The genes were grouped into type I receptors (**A**), type II receptors (**B**), SMADs (**C**), TGF-β superfamily ligands known to mediate SMAD2/3 signaling (**D**), TGF-β superfamily ligands known to mediate SMAD1/5/8 signaling (**E**), and antagonists (**F**). The gene rankings in each group were arranged in an ascending order based on their expression levels. For the groups, such as ligands and antagonists, exhibiting diverse ranges of expression, a TPM cutoff value was set at 0.5 (gray dashed line). For a more complete presentation of the entire profile in these groups, the genes with values less than the cutoff were placed independently in gray dashed boxes. Each gene expression level was expressed as means (black line) ± S.D. n = 18

The diverse signals produced by the TGF-β superfamily dictate the direction of bone homeostasis. To gain a global view of their possible signal intensities in the bone microenvironment, as a first step, we analyzed the expression levels of the various genes involved in TGF-β superfamily signaling module, including surface receptors, intracellular SMADs, extracellular TGF-β superfamily ligands as well as potential antagonists, in a number of available RNA sequencing-based transcriptomic datasets derived from human primary bone cells. In the datasets derived from human normal bone marrows, all genes in the type I receptor, type II receptor and SMAD families can be detected, with *TGFBR1*, *TGFBR2*, and *SMAD3* showing the highest levels of expression in their corresponding family, respectively (Fig. 1A–C and Additional file 2). When analyzing the profiles of TGF-β superfamily ligands involved in conducting SMAD2/3 signaling, we identified that *TGFB1*, encoding the TGF-β1 protein, exhibited an extremely high expression level compared to other ligand genes in the same family (Fig. 1D and Additional file 3); in contrast to this, *BMP6*, *BMP8B*, *BMP2*, *BMP8A* and *AMH* showed relatively high expression levels in the ligand family involved in conducting SMAD1/5/8 signaling (Fig. 1E and Additional file 3). In terms of antagonists, a total of sixteen potential candidates were analyzed and it is noted that the top three genes with the highest expression in bone marrows are *FSTL3*, *TWSG1* and *NOG* (Fig. 1F and Additional file 4).

**Fig. 2:**
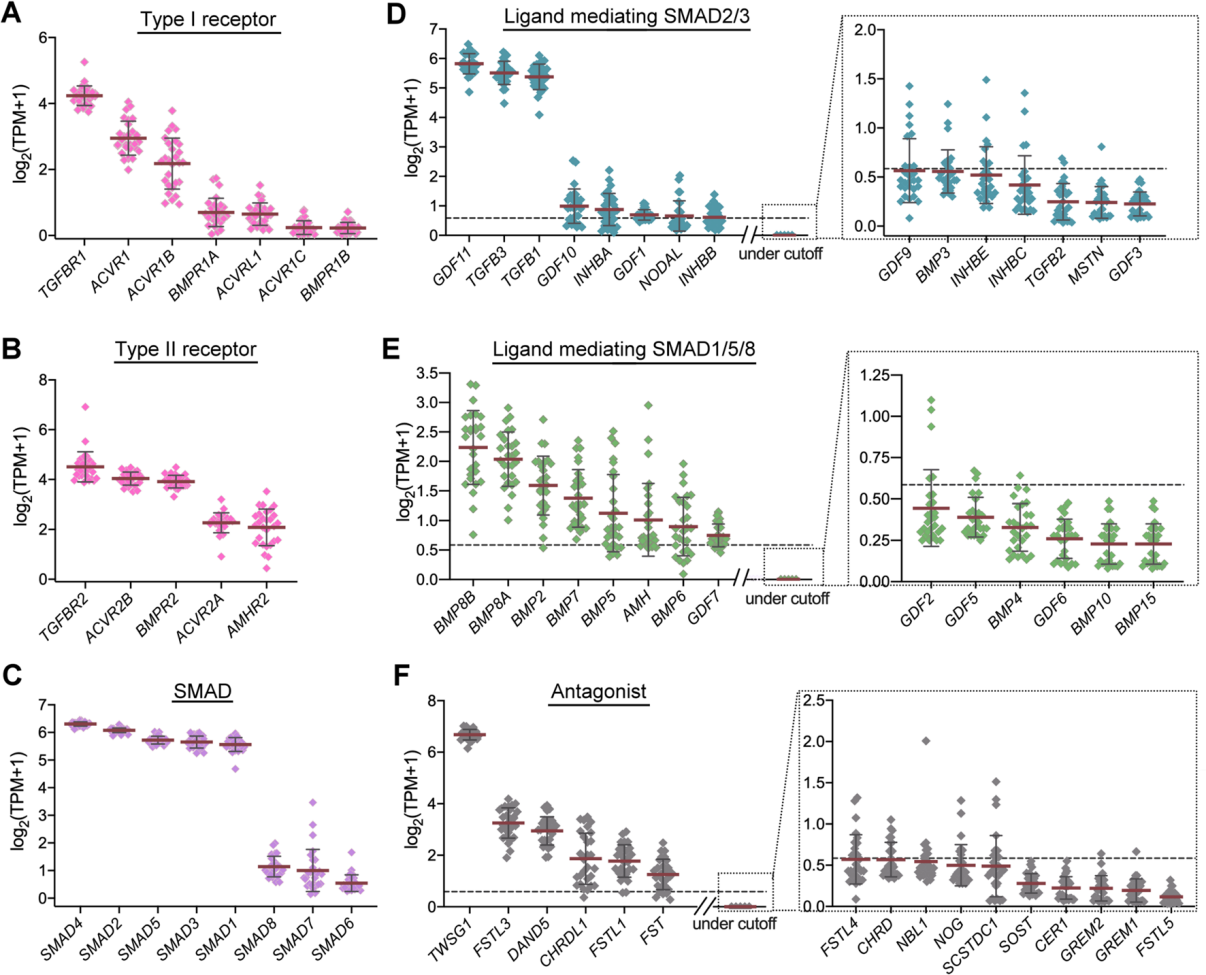
Transcriptomic landscape of the genes in TGF-β superfamily signaling module in human bone marrow-derived MSCs. The transcripts of selected genes were extracted from GSE94736, a transcriptomic dataset from healthy human bone marrow-derived MSCs. The converted TPM values for each gene were presented in Additional files 2 to 4. For the graphic presentation, the data were transformed to log-space by taking log_2_ (TPM + 1) (as described in Materials & Methods). The genes were grouped into type I receptors (**A**), type II receptors (**B**), SMADs (**C**), TGF-β superfamily ligands known to mediate SMAD2/3 signaling (**D**), TGF-β superfamily ligands known to mediate SMAD1/5/8 signaling (**E**), and antagonists (**F**). The gene rankings in each group were arranged in an ascending order based on their expression levels. For the groups, such as ligands and antagonists, exhibiting diverse ranges of expression, a TPM cutoff value was set at 0.5 (gray dashed line). The genes with values less than the cutoff were placed independently in gray dashed boxes. Each gene expression level was expressed as means (black line) ± S.D. n = 28

MSCs play the key roles in determining the rates of bone formation and resorption and also are one of the cell populations that secrete diverse TGF-β superfamily ligands in the bone microenvironment [6, 7]. We thus then analyzed the profiles of TGF-β superfamily signaling-related genes in a dataset derived from primary human bone marrow-derived MSCs. Consistently, the expression of all genes in the receptor families and downstream SMADs can be detected. *TGFBR1* and *TGFBR2* were ranked as the highest expression gene in the type I and the type II receptor families, respectively, while *SMAD4* was ranked first in the SMAD family (Fig. 2A–C and Additional file 2). Notwithstanding the above, there were relatively major differences in the rankings of the TGF-β1 superfamily ligands and antagonists compared to their profiles derived from bone marrows. *GDF11*, *TGFB3* and *TGFB1* were ranked as the top three among the ligands that activate SMAD2/3 signaling, with their expression levels being much higher than the other family members (Fig. 2D and Additional file 3). Among the ligands that conduct SMAD1/5/8 signaling, a number of ligand genes were expressed at the relatively similar level, with *BMP8B*, *BMP8A* and *BMP2* being ranked as the top three (Fig. 2E and Additional file 3). When the antagonists were examined, *TWSG1* exhibited an extremely high expression level compared to all of the other antagonist genes (Fig. 2F and Additional file 4).

### BMP8 activates both SMAD2/3 and SMAD1/5/8 signaling in MSCs

**Fig. 3:**
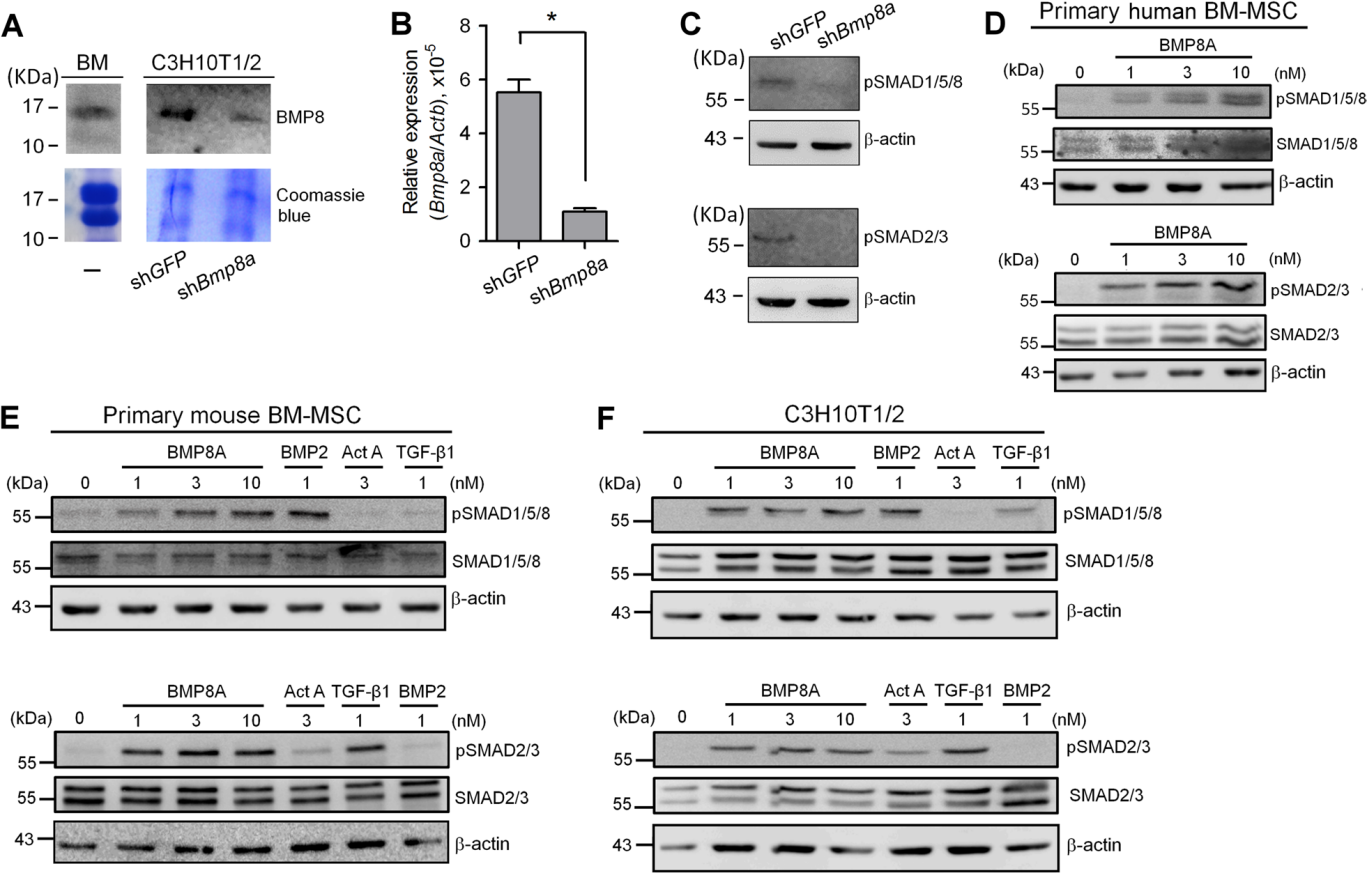
BMP8 activates both SMAD pathways in bone marrow-derived mesenchymal stem cells. **A**–**C** Detection and autocrine function of BMP8 proteins in bone cells. **A** Conditioned media collected from bone marrow (BM) cells or C3H10T1/2 cells without or with *Bmp8a* knockdown were subjected to immunoblotting using the antibody against BMP8. **B** Knockdown efficiency of *Bmp8a* in C3H10T1/2. ***P < 0.001. **C** Knockdown of *Bmp8a* dampens the phosphorylation levels of both SMAD1/5/8 and SMAD2/3 endogenously. To confirm that the BMP8-induced SMAD signaling occurs in MSCs, primary human BM-MSCs (**D**), primary mouse BM-MSCs (**E**) or C3H10T1/2 cells (**F**) were treated with graded doses of BMP8A, BMP2, activin A or TGF-β1 as indicated. The cell lysates were then subjected to immunoblotting using antibody against phosphorylated SMAD1/5/8 (upper panel) or phosphorylated SMAD2/3 (lower panel). Total forms of SMADs and β-actin served as loading controls

Based on the above profiling comparison, we were surprised to find that *BMP8* genes, including *BMP8A* and *BMP8B*, exhibited relatively high expression levels among the ligands involved in SMAD1/5/8 signaling no matter whether the data was derived from bone-marrow cells or bone marrow-derived MSCs (Figs. 1 and 2). In addition to observing this novel finding using human specimens, we here further demonstrated that the BMP8 proteins can be secreted by mouse primary bone-marrow cells and by C3H10T1/2, a mouse MSC line (Fig. 3A). Interestingly, we also observed that knockdown of *Bmp8a* in C3H10T1/2 significantly reduced the phosphorylated levels of both SMAD1/5/8 and SMAD2/3 (Fig. 3B, C), which implies that there is attenuation of both SMAD pathways. Based on this finding, the signals capable of being activated by BMP8 proteins in MSCs were further characterized. Mature human BMP8A and BMP8B proteins differ in only two amino and have been demonstrated to activate similar downstreams [19]. We thus chose only BMP8A for subsequent protein experiments. Surprisingly, contrary to some canonical TGF-β superfamily ligands that selectively activate only one of the SMAD pathways, treatment with BMP8A was found to induce the phosphorylation of both SMAD1/5/8 and SMAD2/3 simultaneously in primary human bone marrow-derived MSCs (Fig. 3D), primary mouse bone marrow-derived MSCs (Fig. 3E), and also C3H10T1/2 cells (Fig. 3F). Due to its abundance and signal uniqueness, we thus speculated that BMP8 would create a yet unknown bio-circuit distinct from other canonical TGF-β superfamily ligands in the bone microenvironment.

### Characterization of the antagonist profiles regulated by the TGF-β superfamily ligands

**Fig. 4:**
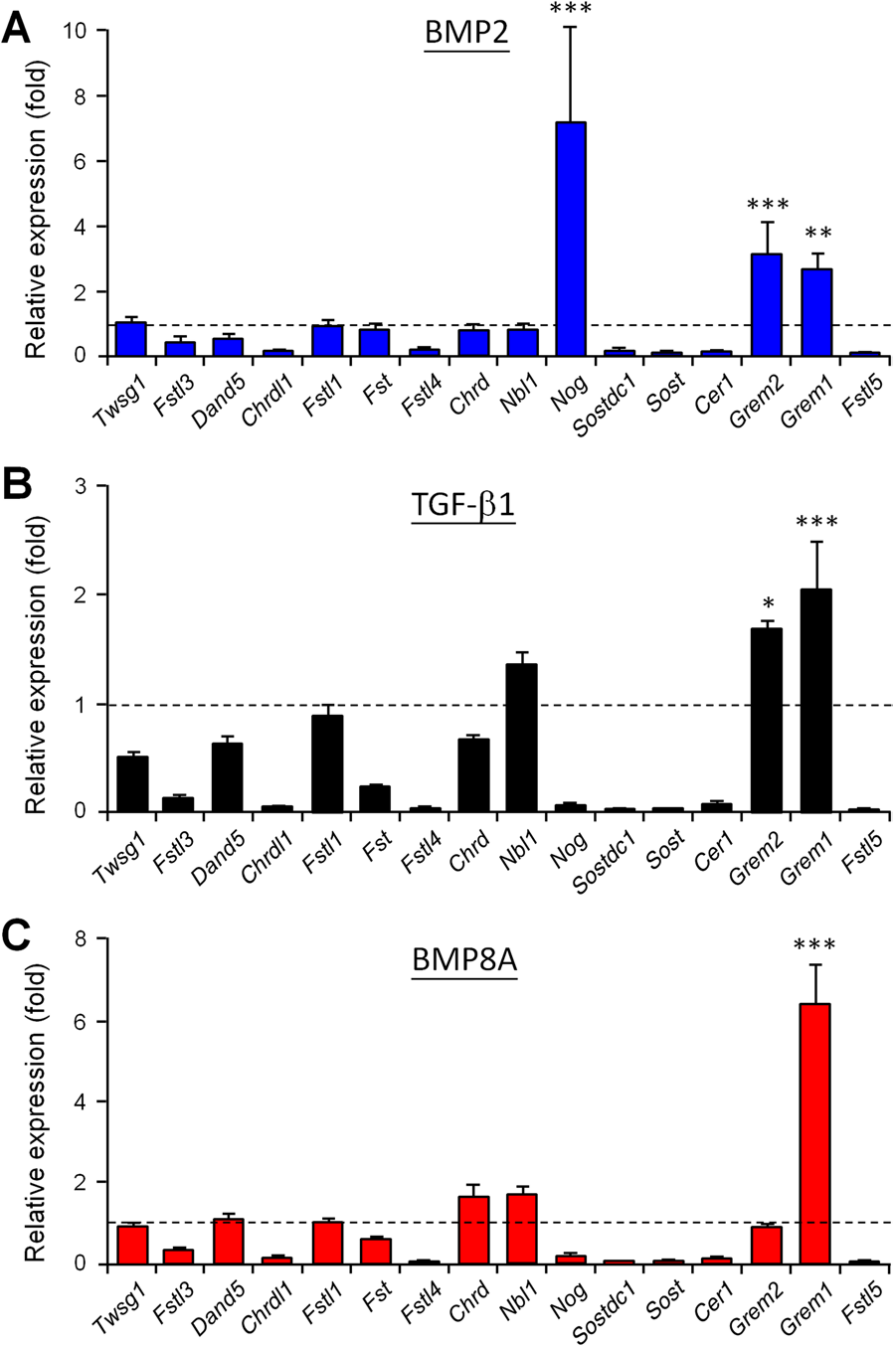
Selected TGF-β superfamily ligands show different effects on the regulation of antagonist expression. C3H10T1/2 cells were treated with 1 nM BMP2 (**A**), 0.3 nM TGF-β1 (**B**) or 1 nM BMP8A (**C**) for 24 h. The transcripts of all TGF-β superfamily antagonists were then quantified. All data were expressed as fold changes by normalizing against the own transcripts of each gene in cells without ligand treatments. Data are shown as means ± SEM. n = 3 independent experiments. *P < 0.05; **P < 0.01; ***P < 0.001

The TGF-β superfamily proteins and their antagonists often act in feedback controls via transcriptional regulation and signaling modulation. We therefore were interested in clarifying their complicated relationships and interactions in MSCs. We firstly explored the changes in antagonist transcriptions in a broad spectrum view under the stimulation of different TGF-β superfamily ligands that are abundantly expressed in the bone microenvironment. Specifically, BMP2, TGF-β1 and BMP8, which showed high levels of expression in our analyzed bone-related transcriptomes, were selected as stimulators for comparison. BMP2 and TGF-β1 are well known stimulators known to activate canonical SMAD1/5/8 and SMAD2/3 signaling in MSCs, respectively; on the other hand, BMP8 can activate both SMAD pathways in MSCs (Fig. 3). Treatment duration was firstly evaluated based on the induction level of *Nog* (Additional file 5), known to be augmented by BMP2 in MSC lineage cells [25]. In the C3H10T1/2 MSC line, treatment with BMP2 for 1 day significantly increased the expression levels of *Grem1*, *Grem2* and *Nog*, with *Nog* showing the highest induction fold (Fig. 4A). By way of contrast, TGF-β1 treatment induced the expression levels of *Grem1* and *Grem2*, but great reduced the expression level of *Nog* (Fig. 4B). BMP8A treatment promoted only the expression of *Grem1*, but did also suppress that of *Nog* (Fig. 4C). In general, all three ligands had the similar effects on *Fstl3*, *Chrdl1*, *Fstl4*, *Sostdc1*, *Sost*, *Cer1* and *Fstl5* in that they reduced the expression levels of these genes by more than half (Fig. 4).

**Fig. 5:**
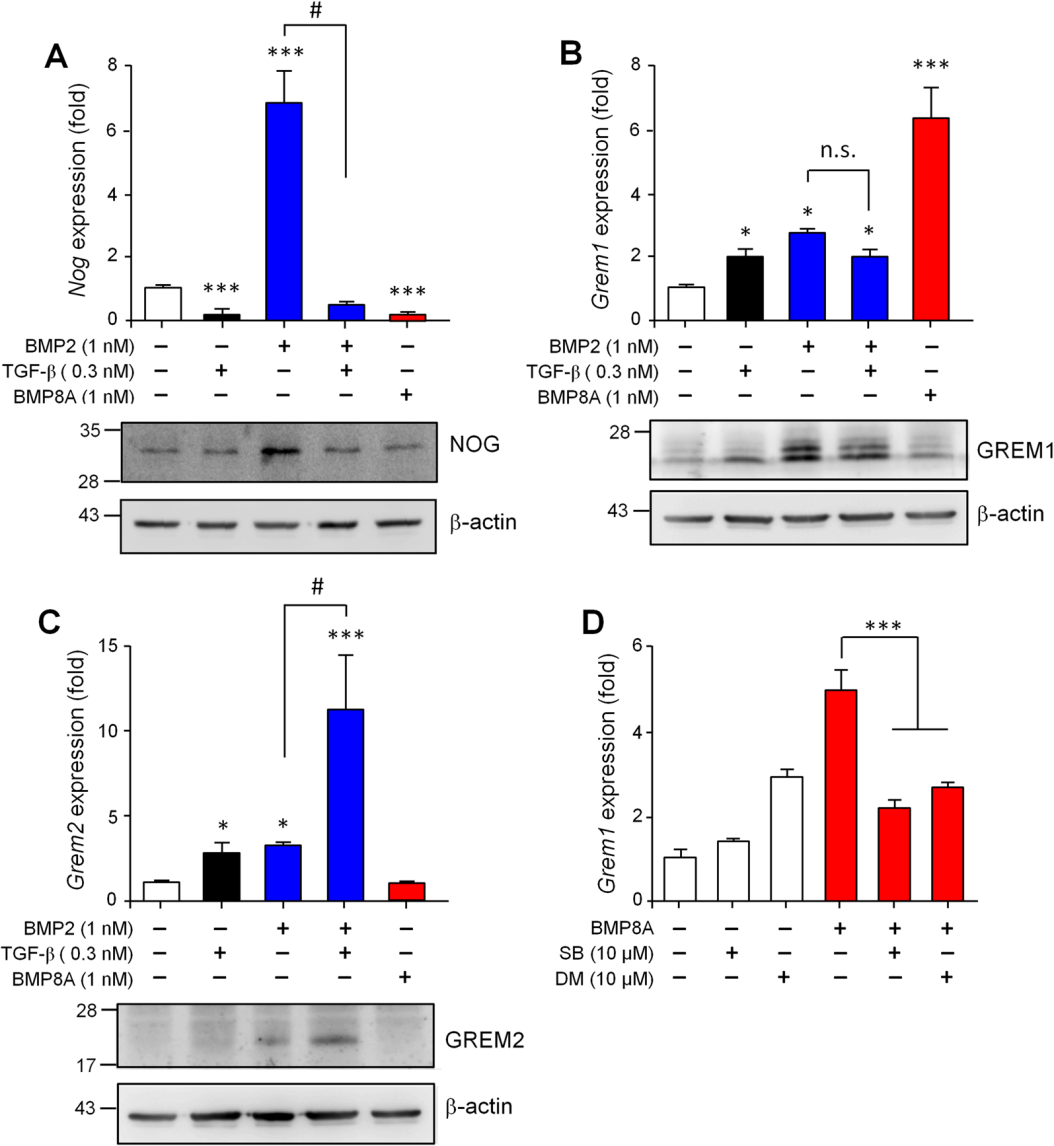
BMP2, TGF-β1 and BMP8A show different effects on the expressional induction of *Nog*, *Grem1* and *Grem2*. C3H10T1/2 cells were treated with BMP2 (1 nM), TGF-β1 (0.3 nM), BMP2 plus with TGF-β1 or BMP8A (1 nM) for 24 h. The transcript levels (upper panel) and encoding protein amounts (lower panel) of *Nog* (**A**), *Grem1* (**B**) and *Grem2* (**C**) were then evaluated. β-actin amounts served as loading controls in immunoblotting. **D** To explore how BMP8A induces *Grem1* expression, cells were treated with BMP8A in the absence or presence of SB431542 (SB) or dorsomorphin (DM) as indicated. Quantification data are presented as means ± SEM. *P < 0.05, ***P < 0.001 compared to no-treatment control. # *P* < 0.001 compared between indicated groups

From the signaling point of view, BMP2, TGF-β1 and BMP8 would represent the activation of different SMAD pathways. We therefore were interested in understanding how a combination of SMAD signaling results in the profiling differences between *Nog*, *Grem1* and *Grem2*. In terms of the *Nog* gene, either TGF-β1 or BMP8A alone can suppress the basal expression level of this gene, while TGF-β1 co-treatment can greatly reverse the BMP2-mediated induction of *Nog* totally (Fig. 5A, upper panel). Similar results were also observed in the immunoblotting analysis by detecting the NOG protein in the lysate of treated cells (Fig. 5A, lower panel). When the *Grem1* gene was investigated, BMP2 and TGF-β1 are both capable of inducing its expression; however, no synergistic promotion was observed co-treatment was carried out. By way of contrast, BMP8A alone augmented the *Grem1* expression to a much higher level than the other two ligands alone and also their co-treatment (Fig. 5B, upper panel). In terms of detection of the GREM1 protein, unlike TGF-β1 and BMP2 treatments, which showed profiles similar to the *Grem1* transcript quantification, BMP8A treatment did not increase the GREM1 protein amount in treated cells (Fig. 5B, lower panel). Furthermore, with the *Grem2* gene, co-treatment with BMP2 and TGF-β1 synergistically promotes its expression in both the mRNA and the protein levels, whereas BMP8A showed no effect (Fig. 5C). Taken together, our findings suggest that these induced antagonists would compose a more complex network in order to modulate the activities of diverse TGF-β1 superfamily ligands.

From the above, we are able to conclude that *Grem1* seems to be the only antagonist induced by BMP8-mediated signaling in MSCs (Figs. 4C and 5). Thus, signaling inhibitors were applied to further explore the possible mechanism(s) involved. We found that administration of either SB431542, an inhibitor of SMAD2/3 signaling, or dorsomorphin, an inhibitor of SMAD1/5/8 signaling, can partially block the BMP8A-induced *Grem1* expression (Fig. 5D), suggesting that both SMAD pathways are involved in this event.

### The induced antagonists counteract the TGF-β superfamily ligands in a selective manner

**Fig. 6:**
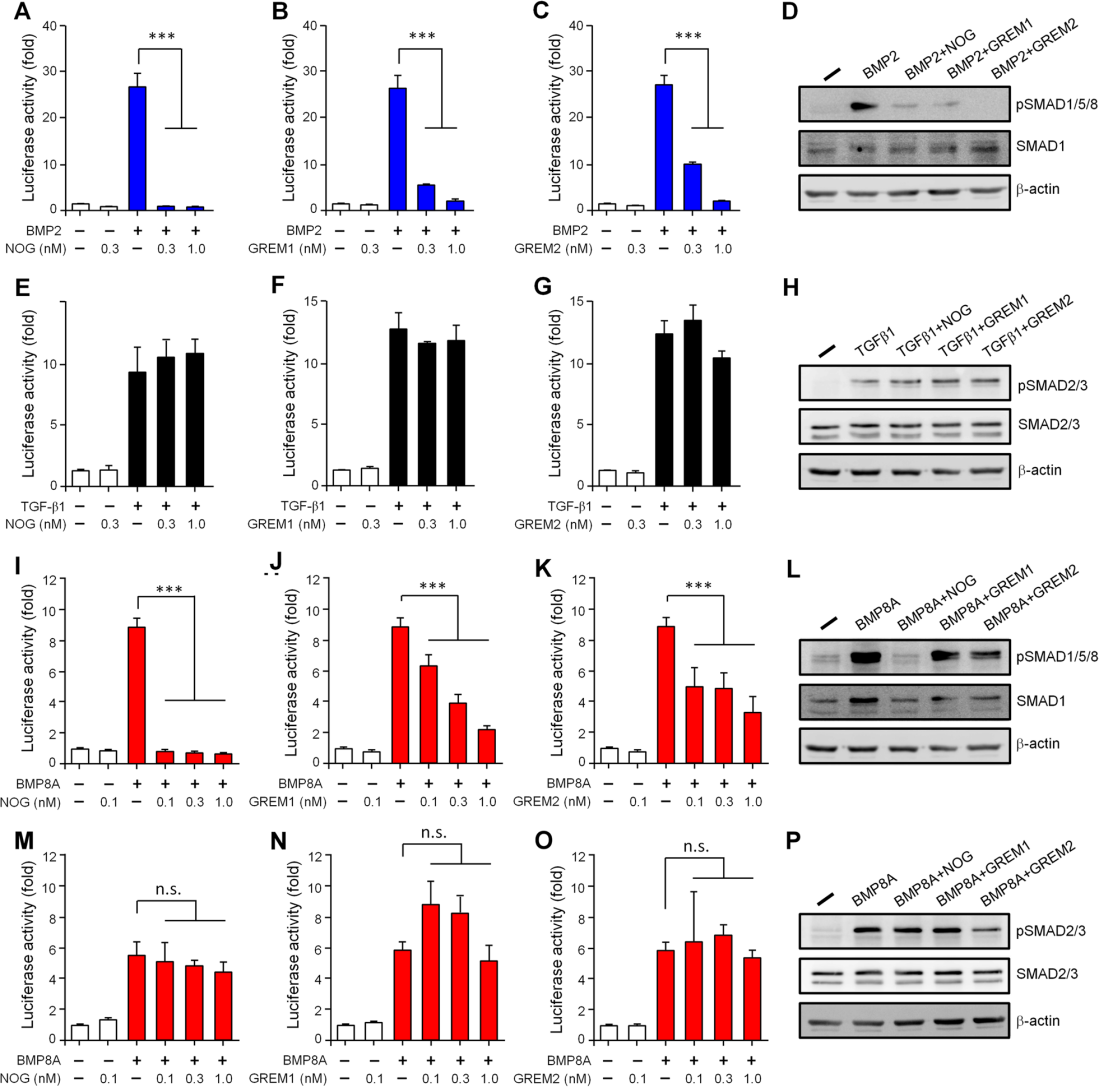
NOG, GREM1, GREM2 have different antagonizing effects on BMP2, TGF-β1 and BMP8A. For testing the antagonisms to BMP2, BRE-Luc reporter-containing HEK293T cells were treated with BMP2 (1 nM) and graded doses of NOG (**A**), GREM1 (**B**) or GREM2 (**C**) as indicated. For testing the antagonisms to TGF-β1, CAGA-Luc reporter-containing HEK293T cells were treated with TGF-β1 (0.3 nM) and graded doses of NOG (**E**), GREM1 (**F**) or GREM2 (**G**) as indicated. For testing the antagonisms to BMP8A, BRE-Luc reporter-containing cells (**I**–**K**) or CAGA-Luc reporter-containing cells (**M**–**O**) were treated with BMP8A (1 nM) and graded doses of NOG (**I** and **M**), GREM1 (**J** and **N**) or GREM2 (**K** and **O**) as indicated. After treated overnight, reporter activity in the cells was measured. The luciferase data were normalized against the β-galactosidase levels to correct for transfection efficiency and were expressed as fold changes by normalizing against the values obtained from control cells without treatment. Data are expressed as the mean ± SEM. n = 3. ***P < 0.001. For monitoring the phosphorylated SMAD levels, HEK293T cells were treated with BMP2 (**D**), TGF-β1 (**H**) or BMP8A (**L** and **P**) in the absence or presence of indicated antagonists (1 nM) for 30 min. Cell lysates were subjected to immunoblotting to detect the phosphorylated SMADs. Total forms of SMADs and β-actin served as loading controls

By characterizing the changes in the whole antagonist profiles above (Fig. 4), we were surprised to find that only the expressions of *Nog*, *Grem1* and *Grem2* can be induced by the abundant TGF-β superfamily ligands present in MSCs. We therefore were interested in exploring whether these induced antagonists provide a negative-feedback loop that can hamper the activity of these TGF-β superfamily ligands. We found that NOG, GREM1 or GREM2 can effectively antagonize the BMP2-induced SMAD1/5/8 signaling (Fig. 6A–D), but they have no effect on blocking the TGF-β1-induced SMAD2/3 signaling (Fig. 6E–H). When BMP8A was explored, NOG exhibited strong antagonism, but GREM1 and GREM2 showed only moderate inhibition, to BMP8A-activated SMAD1/5/8 signaling (Fig. 6I–L); surprisingly, they all had no effect on BMP8A-activated SMAD2/3 signaling (Fig. 6M–P). Thus, in agreement with their expression profiles and antagonizing effects, these induced antagonists in the bone microenvironment would not only provide negative feedback loops toward specific TGF-β superfamily ligands, but also fine-tune the preference toward the different SMAD pathways that are induced by the same ligand.

### Characterization of the expressional changes of TGF-β superfamily signaling module in primary osteoporosis and the involvement of functional networks

**Fig. 7:**
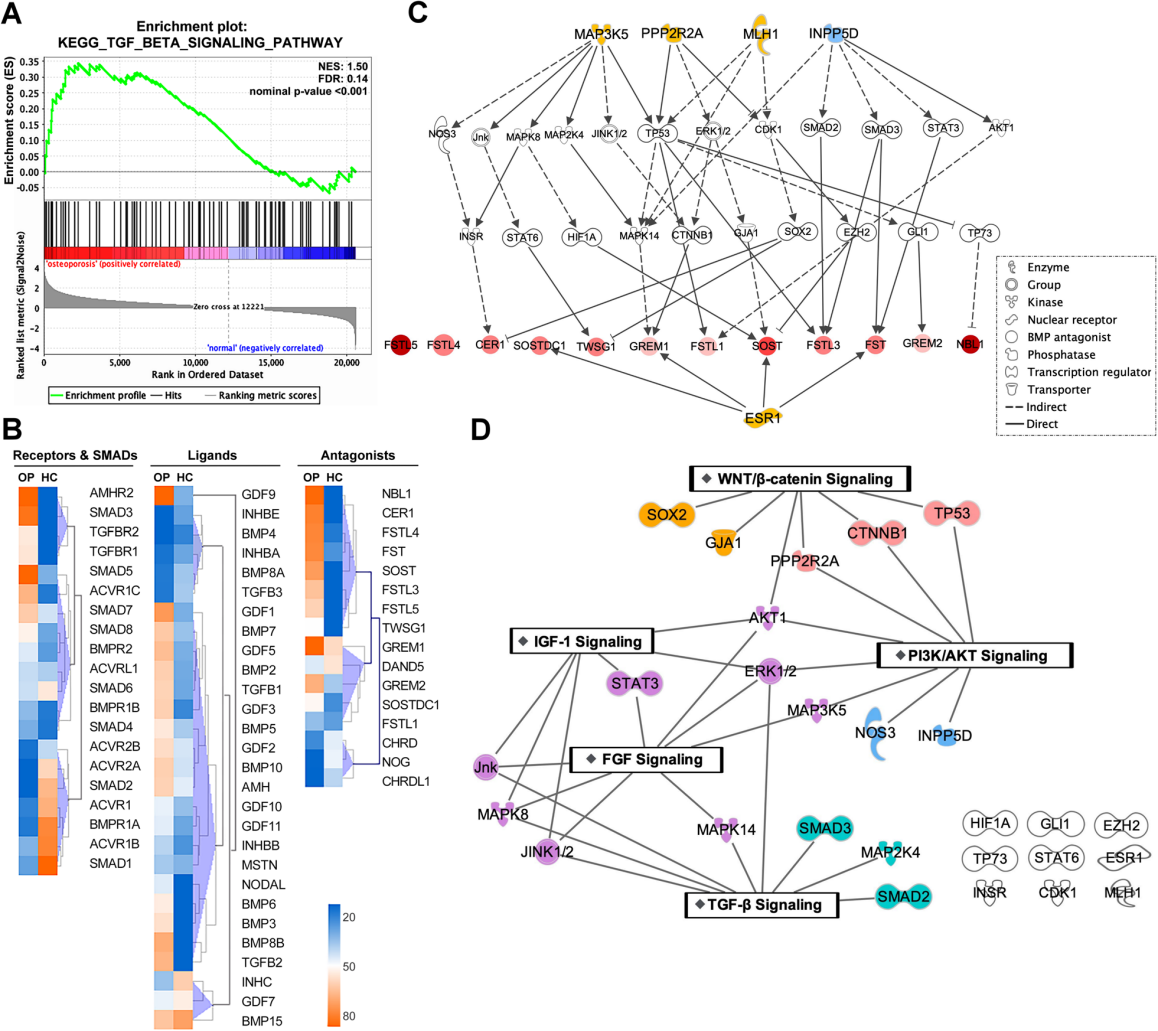
Differential expression of the genes in TGF-β superfamily signaling module in osteoporotic MSCs and their potential network analysis. **A** The enrichment plot computed by GSEA indicated the TGF-β signaling gene set was significantly enriched in bone marrow-derived MSCs from elderly patients suffering from primary osteoporosis. **B** The relative expressions of each gene in TGF-β superfamily signaling module between MSCs from patients suffering from primary osteoporosis (OP) and their age-matched healthy controls (HC) were compared in a heat‐map manner. The profiles were divided into three groups, membrane receptors and SMADs (left), ligands (middle) and antagonists (right). Expression patterns were clustered by Manhattan distance calculation. **C** Causal network analysis identified 27 regulators potentially promoting the expression of the 12 selected BMP antagonists. The network was constructed by setting depth up to 3 (2 intervening regulators). Yellow pattern: activation (Z-score > 2); blue pattern: inhibition (Z-score < −2). Patterns with gradient color from light to dull red reflected the enhanced transcription folds of BMP antagonists showed in the microarray data. **D** IPA analysis of the possible canonical signaling pathways connected with the identified regulators

Primary osteoporosis is the most common form of osteoporosis characterized by age-related low bone mass and microarchitectural deteriorations [26]. We therefore were interested in exploring whether the genes in TGF-β superfamily signaling module are regulated and/or involved in this bone disease model. We here analyzed GSE35958 [27], a transcriptomic dataset of bone marrow-derived MSCs from elderly patients suffering from primary osteoporosis (79–94 years old; n = 4) and age-matched controls (79–89 years old; n = 4). To investigate the potentially altered pathways, the normalized data were further processed by GSEA using the gene sets of canonical pathways derived from the KEGG pathway database (C2-CP-KEGG). As expected, we did identify that TGF-β superfamily signaling pathway is enriched in primary osteoporosis sets (normalized enriched score (NES) = 1.50; false discovery rate (FDR) = 0.14) (Fig. 7A).

We then sought to comprehensively profile the expressional changes of the genes in this signaling module. In the genes of membrane receptors and downstream SMADs, hierarchical clustering analysis indicates that these genes could be roughly divided into three subclusters (Fig. 7B, left panel). Such clustering results are interesting as *TGFBR1*, *TGFBR2* and *SMAD3* formed a cluster that showed enhanced expression in primary osteoporosis. These three genes are all required for conducting TGF-β-mediated SMAD3 signaling and also showed relatively high basal expression levels in the families to which they belong in human primary MSCs (Fig. 2). By way of contrast, the cluster enriched in healthy controls contained more genes required for conducting SMAD1/5/8 signaling, such as *ACVR2A*, *ACVR1*, *BMPR1A* and *SMAD1*. Although we did not observe strong correlation between the specific ligand population and osteoporosis in the ligand profiles (Fig. 7B, middle panel), the above differential expression trends suggest that the seesaw balance of SMAD pathways would lean to over-activation of TGF-β-mediated SMAD3 signaling in MSCs of osteoporosis. Since we have demonstrated that activation of SMAD2/3 signaling can effectively reverse the induction effect of BMPs on *NOG* expression in MSCs (Fig. 5A), the above conclusion would also partially explain the apparent suppression of *NOG* expression in osteoporotic MSCs when the antagonist profiles were compared (Fig. 7B, right panel). Consistent with the above, in MSCs of primary osteoporosis we also observed the enhanced expression of *GREM1* and *GREM2*, the only two antagonist genes found to be commonly up-regulated by SMAD signaling in this study (Figs. 4 and 5). Intriguingly, unlike genes in receptors and ligands, we further noticed that twelve out of sixteen antagonist genes showed enhanced expression in MSCs of primary osteoporosis in comparison to their age-matched controls (Fig. 7B, right panel); these include those genes supposed to be down-regulated by SMAD signaling in our study, such as *Fstl3*, *Fstl4*, *Fstl5*, *Sostdc1*, *Sost* and *Cer1* (Fig. 4). The general augmentation of antagonist genes not only reflects intrinsic attenuation in BMP-mediated osteogenesis but also implies that other signaling pathways are involved in the transcriptional induction of these genes during osteoporosis.

Subsequently, the twelve up-regulated antagonist genes were subjected to IPA to estimate the possible functional networks. Causal network analysis allowed 27 regulators to be identified for the possible up-regulation of these twelve antagonist genes (Fig. 7C). Rather interestingly, hierarchical relationship indicates that *MAP3K5*, *MLH1* and two phosphatase-related genes, *PPP2R2A* and *INPP5D*, were found to be critical master regulators of 22 intermediate regulator genes, whereas expression of *ESR1* can directly up-regulate four antagonist genes. We then uploaded these 27 regulators into IPA for overlaying the connection with canonical pathways and the results suggest that at least five signaling pathways, including FGF signaling, PI3K/AKT signaling, TGF-β signaling, Wnt/β-catenin signaling and IGF-1 signaling, would work synergistically for the general induction of these antagonist genes (Fig. 7D).

## Discussion

TGF-β superfamily signaling has fundamental roles in bone homeostasis. Distinct from most of the previous studies that have characterized the profiles and roles of individual genes involved in the TGF-β superfamily signaling, our study for the first time provides a global view of the expression profiles of most known genes in this module in the bone microenvironment and their temporal changes in MSCs after the occurrence of primary osteoporosis. In the bone microenvironment, we have noticed that some genes show different rankings in terms of their expression levels between bone marrow cells and bone marrow-derived MSCs. The differences may be explained by the fact that the cells other than MSCs are present in the bone marrows, such as cells in the hematopoietic lineage and differentiated cells in the osteochondrogenic lineage.

In the bone marrow cells, we identified that *TGFB1* has the highest transcript level among the group of ligands capable of activating SMAD2/3 signaling. Consistently, via protein isolation and characterization, TGF-β proteins have been identified as the most abundant cytokines in the bone matrix (200 μg/kg) [9, 10]. In terms of the ligands that mediate SMAD1/5/8 signaling, our transcriptomic analysis results indicate that *BMP6*, *BMP2*, *BMP8A and BMP8B* are abundantly expressed in the human bone niches. Interestingly, although *BMP6* was identified to be the most abundant BMP in bone marrow cells, mice with conventional knockout of *Bmp6* show no distinguishable phenotype with regard to their bones, except that there is a slight delay in ossification in the developing sternumin [28]. Although species difference may be considered, these studies also imply that these BMPs may functionally compensate for each other due to their co-expression in the bone microenvironment. If this hypothesis is true, the bone-related phenotypes should be highlighted when multiple members of these functional redundant genes are depleted. Taking BMP2 and its evolutionarily close paralog BMP4 as examples, conditional loss of either *Bmp2* or *Bmp4* alone in the mouse limb bud mesenchyme does not affect limb skeletogenesis [29, 30]; however, conditional deletion of both *Bmp2* and *Bmp4* in the mouse limb bud mesenchyme indeed appears a severe impairment of limb osteogenesis [31]. Regarding BMP8, we previously have demonstrated that BMP8 is abundantly expressed in the reproductive system and can affect the postnatal spermatogenesis and ovarian folliculogenesis via its unique ability to activate both SMAD1/5/8 and SMAD2/3 pathways simultaneously [19, 32, 33]. In terms of its functions in bone, although not yet being well clarified, several clues have linked BMP8 with bone homeostasis. For example, the *Bmp8* gene is highly expressed in the developing skeletal tissues of mouse embryos [34]. The BMP8 proteins seem to be induced in the healing region of the fractured bones in mice at the periods when the resorption of calcified cartilage and osteoblastic recruitment are most active [35, 36]. Further, using the cultured mouse cell models, it has been reported that BMP8 proteins can be produced by osteoblasts and have a protective role in the glucocorticoid-induced cell death via an autocrine loop [37]. These studies support that *Bmp8* can be expressed and would have a crucial role in murine bone; however, there is no report up to date about the expression and function of *BMP8* in humans. With the abundant expression in the human bone niches found in our study here, it would be interesting to explore whether and how BMP8 affects human bone homeostasis and bone-related diseases via its unique signaling.

Based on our transcriptomic analyses regarding the TGF-β superfamily antagonists, it can be concluded that *FSTL3* and *TWSG1* are the most abundant antagonists expressed in the mammalian bone niches (Figs. 1F and 2F); however, their expression levels in MSCs do not seem to be induced by the signaling activated by the TGF-β superfamily ligands (Fig. 4). Notwithstanding the above, we identified that *Nog*, *Grem1* and *Grem2* are the only three antagonist genes capable of being induced by SMAD signaling. Via literature reviews, it seems that these induced antagonists do have profound effects on the bone development. Specifically, *NOG*, the most abundant candidate among these three induced antagonist genes in the human bone niches (Figs. 1F and 2F), has been found to play important roles in bone homeostasis since mice with either depletion or overexpression of *Nog* show strong skeletal phenotypes during development. Knockout of *Nog* results in oversized growth plates and joint lesions in mice [38]; these limb phenotypes are similar to the effects resulted from overexpression of BMPs in the chick models [39, 40]. By way of contrast, transgenic mice with skeletal overexpression of *Nog* show impaired osteoblastic function that leads to osteopenia and fractures [41]. Of interest, some of the above phenotypes are also observed in mice with *Grem1* gene manipulation. In mice, conditional deletion of *Grem1* in osteogenic linage cells enhances osteoblastic activity and bone formation [42], whereas skeletal overexpression of *Grem1* decreases the number and function of osteoblasts, leading to osteopenia and spontaneous fractures [43]. Contrary to the above, *Grem2*^−/−^ mice show only deformed incisor teeth and minor elevation of bone mineral density [44], suggesting that GREM2 is not vital to skeletal development, or that its functional loss can be compensated by GREM1.

Since the antagonists dampen the signaling of TGF-β superfamily ligands by direct binding with these ligands and precluding their interaction with receptors [14], the above phenotypes observed in mice with *Nog* or *Grem1* manipulation may be indirectly mediated through a shift in the signaling intensity of specific TGF-β superfamily ligands due to the breakdown of antagonist feedback loop to specific ligands. Such a balance between TGF-β superfamily ligands and their corresponding antagonists may further explain why the skeletal functions of some TGF-β superfamily ligands behave differently when in vitro experiments and in vivo conditions are compared. Taking TGF-βs as an example, although they exhibit the ability to inhibit BMP-mediated osteoblast maturation and mineralization in vitro [13], TGF-βs seem to enhance BMP-induced bone formation in vivo [45, 46]. This might be due, at least in part, to the fact that TGF-βs strongly suppress *Nog* expression in the bone niches (Fig. 5), thus promoting the BMP activity in vivo by reversing the NOG-mediated negative-feedback loop induced by BMPs [47].

In this study, we also observed that the effects of BMP8 proteins on the expression of *Nog*, *Grem1* and *Grem2* are quite distinct from those of BMP2, TGF-β1 or BMP2 in combination with TGF-β1 (Fig. 5). As to *Nog*, it seems that only the downstream of SMAD2/3 signaling, either activated by TGF-β1 or BMP8, is enough to suppress its expression induced by BMP2. However, although activating both SMAD1/5/8 and SMAD2/3 signaling in MSCs, BMP8 showed different regulatory impacts to *Grem1* and *Grem2* as compared to the co-treatment with BMP2 and TGF-β1. This may be explained by differences in the receptor population used by these ligands. Our previous study has suggested that BMP8 seems to exhibit a greater flexibility in terms of receptor selection [19]. Taking the SMAD2/3 activation as an example, TGF-β1 strictly binds to the receptor complex formed by TGFBR1 and TGFBR2 [48], whereas BMP8 may interact with the receptor complexes formed by the type I receptor ACVR1B or TGFBR1 and the type II receptor ACVR2A, ACVR2B, or TGFBR2 [19]. Thus, the difference in the mediating receptors may allow TGF-β1 and BMP8 to induce different populations of transcriptional regulators, leading to the different regulatory impacts on *Grem1* and *Grem2*. Intriguingly, the above findings not only address the need for antagonist feedback mechanisms that balance the signal intensity of TGF-β superfamily ligands, but also point out the possible application of these molecules during bone regeneration. BMP2 is currently a FDA-approved osteoinductive drug [49]; however, it strongly triggers the NOG feedback loop that limits the BMP activity [25]. This partially explains why high doses are needed to reach clinical efficacy (~ 1.5 mg/ml in the anterior interbody spine fusion for example) [50]. A later study further proposes that co-treatment with TGF-βs can effectively suppress the BMP-induced *Nog* expression and thus may enhance the BMP-mediated bone fracture-healing process [47]. Although this hypothesis seems to be partially true in treated MSCs shown in our findings (Fig. 5A), we further found that both BMP2 and TGF-β1 can individually induce expression of *Grem1* and *Grem2* and that they work even better to synergistically promote *Grem2* expression (Fig. 5B, C). On the other hand, BMP8 strongly suppresses *Nog* expression and induces only *Grem1* expression. Thus, with a minimal augmentation of the antagonist feedback loop, BMP8 alone would replace the co-treatment with BMP2 and TGF-β1, and may even require a lower dose to reach the similar clinical efficacy when applied to the orthopaedic surgery.

We here further identified that TGF-β superfamily signaling module showed distinct expressional changes in MSCs from age-related osteoporotic patients. Especially, the expressions of most antagonist genes were significantly enhanced (Fig. 7B). By computing the possible networks, at least five signaling pathways were predicted to work synergistically for the induction of these antagonist genes. Indeed, by the strategies such as gene manipulation and genetic analysis, some factors critical for these pathways, such as FGF23 and Klotho in FGF signaling [51, 52], WNT3A and LRP5 in Wnt signaling [53, 54], IGF-1, IRS-1 and IRS-2 in IGF-1 signaling and downstream PI3K/AKT pathway [55–57], have been found to be relevant to osteoporosis. Notwithstanding the above, the crosstalks among these signaling pathways have been rarely explored. Our study here established the causal networks to elucidate how these signaling pathways interact coordinately to modulate the expressions of the antagonist genes in TGF-β superfamily signaling module.

## Conclusions

In summary, our studies for the first time unveiled the transcription landscape of all the genes in TGF-β superfamily signaling module in the bone microenvironment. Such global views allowed us to clarify the interplays between TGF-β superfamily ligands and their induced antagonists in bone of normal humans and to propose the therapeutic potential of BMP8 in orthopaedic surgery. Further, the differential expression of these genes in MSCs of primary osteoporotic patients not only demonstrated that blocking BMP signaling via general augmentation of the antagonist expression levels is an important feature of primary osteoporosis, but also provided a different hint and concern when developing strategies to treat osteoporosis.

## Supplementary Information

**Additional file 1**. Table S1. Primers used for qPCR.

**Additional file 2**. Table S2. Expressional analysis and relative ranking of TGF-β superfamily receptors and SMADs in bone-related transcriptomes.

**Additional file 3**. Table S3. Expressional analysis and relative ranking of TGF-β superfamily ligands in bone-related transcriptomes.

**Additional file 4**. Table S4. Expressional analysis and relative ranking of TGF-β superfamily antagonists in bone-related transcriptomes.

**Additional file 5**. Figure S1 Induction effect of BMP2 on Nog expression. C3H10T1/2 cells were treated without or with BMP2 (1 nM) for different intervals. The cells were then harvested and subjected to quantify the transcript of *Nog*. The values at the BMP2 treatment group were first normalized with those at the control cells harvested at the same time point and were expressed as fold changes. Data are expressed as the means ± SD. n=3. ***P < 0.001.

## Data Availability

All data generated or analyzed during this study are included within the article and its additional files.

## Abbreviation

IPA: Ingenuity Pathway Analysis
MSC: Mesenchymal stem cell
TPM: Transcripts per million
