# Supplementary material for: Unveiling the transcriptomic landscape and the potential antagonist feedback mechanisms of TGF-β superfamily signaling module in bone and osteoporosis
Source: Cell Commun Signal. 2022 Nov 28;20:190. doi: 10.1186/s12964-022-01002-2 (PMC9703672; doi:10.1186/s12964-022-01002-2)
Supplement: Supplementary file 2 — Additional file 1. Table S1. Primers used for qPCR. [file 12964_2022_1002_MOESM2_ESM.docx]

**Table S1.** Primers used for qPCR.

| **Gene**  **(rat)** | **Accession no.** | **Forward primer**  **(5’→ 3’)** | **Reverse primer**  **(5’→ 3’)** | **Amplicon** |
| --- | --- | --- | --- | --- |
| *Fst* | NM_001301373.1 | AACCCCgCTgCgTCTgT | ACATTCgTTgCggTAggTTTTC | 92 |
| *Fstl1* | NM_008047.5 | TCCTCCATCggCAACCAA | CAgAAgCACAgCAAATACAAATgC | 145 |
| *Fstl3* | NM_031380.2 | CAgggCACAgCCgATgA | TgCCCgCTCTggTCTgA | 58 |
| *Fstl4* | NM_177059.3 | gCTTCATCgTCAgTgTCTCCAA | gATTTCCCCCCgCACTgT | 71 |
| *Fstl5* | NM_178673.4 | CgCCAAATCCTgCCTTCA | CTTCCTgTCCATTTTCTATAgCACAA | 145 |
| *Twsg1* | NM_023053.3 | CACCgTgAgCACCAgCAA | CTgCCCATAAgCCCATgTCT | 104 |
| *Sost* | NM_024449.6 | TCTACCCTCgCCggACCT | TCCATAACCAgTCCCAggTCTT | 91 |
| *Sostdc1* | NM_025312.3 | TAAACCCCTTCCCAAACTAAATCC | CTAACCCCTggCTgTCAAACTATT | 130 |
| *Chrd* | NM_009893.2 | gCACCCAACTATgACCCACTCT | TgCgTTgTTTCTCTggACACA | 148 |
| *Chrdl1* | NM_001114385.1 | ACTTCTTgCCAgCCAATCACA | AgggAAgCAgCAgAggTTTg | 121 |
| *Cer1* | NM_009887.2 | AggTTCTggCATCggTTCAT | ggCTTTTgATgggCAggAT | 70 |
| *Nog* | NM_008711.2 | CCAgCACCCAgCgACAAC | ggTCAAAgATAgggTCTggATgTT | 64 |
| *Grem1* | NM_011824.4 | CCTggAgAggAggTgCTTgA | TTgATgATAgTgCggCTgTTg | 140 |
| *Grem2* | NM_011825.1 | CAAAgAATAgACTCCTAAAATACCAAgAAA | AAgACCACACAACAgAgACTCCAA | 89 |
| *Nbl1* | NM_008675.2 | gCCCCgCCACCTATCAA | ggCTTCACACCAggCACTCT | 60 |
| *Dand5* | NM_201227.3 | TCggCgggTgAggATTT | CTTCgggCggCACTgA | 55 |
| *Bmp8a* | NM_001256019 | CAgCCCACAATggCAAATT | AATggTgCAgAgAgCCCAgAT | 80 |
| *Actb* | NM_007393.5 | CTCTgTgTggATCggTggCT | TgCTTgCTgATCCACATCTg | 69 |
