# Supplementary figures and images for: Unveiling the transcriptomic landscape and the potential antagonist feedback mechanisms of TGF-β superfamily signaling module in bone and osteoporosis
Source: Cell Commun Signal. 2022 Nov 28;20:190. doi: 10.1186/s12964-022-01002-2 (PMC9703672; doi:10.1186/s12964-022-01002-2)

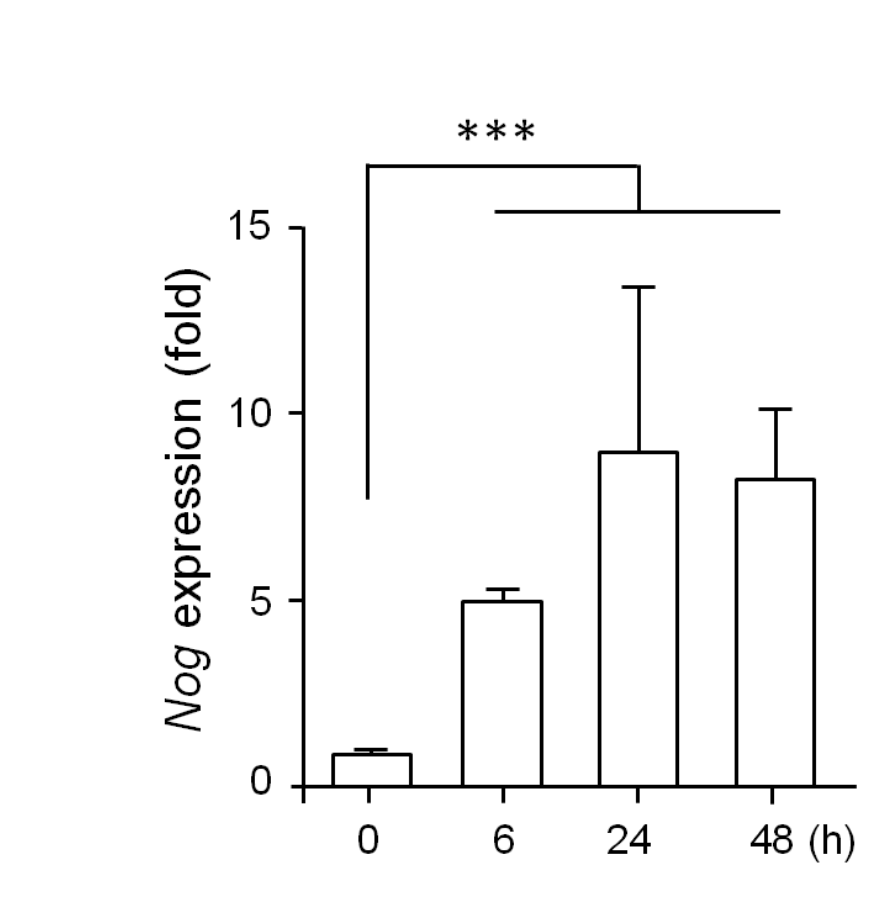

Supplement: Supplementary file 6 — Additional file 5. Figure S1 Induction effect of BMP2 on Nog expression. C3H10T1/2 cells were treated without or with BMP2 (1 nM) for different intervals. The cells were then harvested and subjected to quantify the transcript of Nog. The values at the BMP2 treatment group were first normalized with those at the control cells harvested at the same time point and were expressed as fold changes. Data are expressed as the means ± SD. n=3. ***P < 0.001. [file 12964_2022_1002_MOESM6_ESM.tif]
